# Early postpartum abnormal glucose metabolism subtype differs according to mid-trimester lipid profile in women with gestational diabetes mellitus

**DOI:** 10.1186/s12944-021-01519-4

**Published:** 2021-08-24

**Authors:** Fenghua Lai, Zeting Li, Shufan Yue, Ling Pei, Huangmeng Xiao, Zhuyu Li, Yanbing Li, Haipeng Xiao, Xiaopei Cao

**Affiliations:** 1 Department of Endocrinology, The First Affiliated Hospital, Sun Yat-sen University, 58 Zhongshan 2nd Rd, 510080 Guangzhou, China; 2 Department of Obstetrics and Gynecology, The First Affiliated Hospital, Sun Yat-sen University, 510080 Guangzhou, China

**Keywords:** Gestational diabetes mellitus, Lipid profile, Mid-trimester, Glucose metabolism, Postpartum

## Abstract

**Background:**

It is unknown whether early postpartum abnormal glucose metabolism (AGM) in women with previous gestational diabetes mellitus (GDM) is related to their mid-trimester lipid profile. The aim of this study was to characterize the mid-trimester lipid profile of women who experienced GDM and developed into different pathophysiologic subtypes of early postpartum AGM.

**Methods:**

A retrospective cohort study of 498 women with history of GDM was conducted. A 75-g oral glucose tolerance test (OGTT) and plasma lipid measurements were performed at 24–28 weeks of gestation and 6–12 weeks of postpartum. Insulin secretion and sensitivity were estimated using early postpartum OGTT-based indices.

**Results:**

Women in the mid-trimester dyslipidemia group had higher postpartum 30-min and 2-h plasma glucose, higher postpartum 2-h plasma insulin, higher postpartum triglyceride (TG), higher postpartum low density lipoprotein cholesterol (LDL-c) concentrations, lower postpartum 30-min insulinogenic index (IGI_30_), lower postpartum insulin sensitivity index (ISI), and lower postpartum disposition index than those in the normal lipid group (all *P* < 0.05). Abnormal mid-trimester TG and LDL-c concentrations were associated with postpartum AGM (adjusted odds ratio [OR] = 1.786, 95 % confidence interval [CI] = 1.142–2.425; and adjusted OR = 1.621, 95 % CI = 1.323–2.051, respectively; both *P* < 0.05). AGM women with low IGI_30_ and low ISI had higher mid-trimester total cholesterol and LDL-c concentrations, and AGM women with low ISI had higher mid-trimester TG concentrations than women with NGT or other subtypes of AGM (all *P* < 0.05).

**Conclusions:**

GDM women with abnormal mid-trimester TG and LDL-c were predisposed to early postpartum AGM. Postpartum AGM women who experienced GDM had heterogeneous mid-trimester lipid profile when classified according to their pathophysiologic subtype.

## Background

Gestational diabetes mellitus (GDM) is a common complication of pregnancy that affects approximately 18 % of pregnancies per year worldwide [1]. Insulin resistance, a main factor in GDM pathophysiology, is associated with a more atherogenic lipid profile. The high-fat diet, weight gain, and increased body mass index are key risk factors of developing GDM [2]. GDM not only increases the risk of GDM in future pregnancies [3], but also increases the risk of postpartum type 2 diabetes mellitus (T2DM) [4]. The American Diabetes Association recommended that a 75-g oral glucose tolerance test (OGTT) should be performed at 4–12 weeks of postpartum in every woman who experienced GDM [5]. However, the rate of attendance was still low [6]. The early diagnosis of postpartum prediabetes can provide an opportunity to reduce the risk of T2DM through interventions comprising appropriate lifestyle modifications. Therefore, the early identification of biomarkers associated with postpartum abnormal glucose metabolism (AGM) should promote health and help prevent subsequent T2DM.

Pregnancy substantially alters lipid metabolism. Previous studies showed that pregnancies with GDM were characterized by more severe hyperlipidemia than pregnancies with normal glucose tolerance (NGT) [7]. Dyslipidemia in pregnancy were associated with pregnancy complications, such as pre-eclampsia, pre-term birth, and postpartum hypertension in the longer term [8, 9]. Furthermore, a previous case-control study demonstrated that GDM pathophysiologic subtype differed in their gestational lipid profiles, which emphasized the pathophysiologic heterogeneity of GDM [10]. However, it is unknown whether early postpartum AGM in women who have experienced GDM differs according to their gestational lipid profile.

Therefore, a retrospective cohort study to explore the relationship between lipid profile during the mid-trimester and postpartum AGM in women who had previously experienced GDM was conducted. Furthermore, the mid-trimester lipid profile of the women was analyzed according to their pathophysiologic subtype of early postpartum AGM.

## Methods

### Study participants

**Fig. 1:**
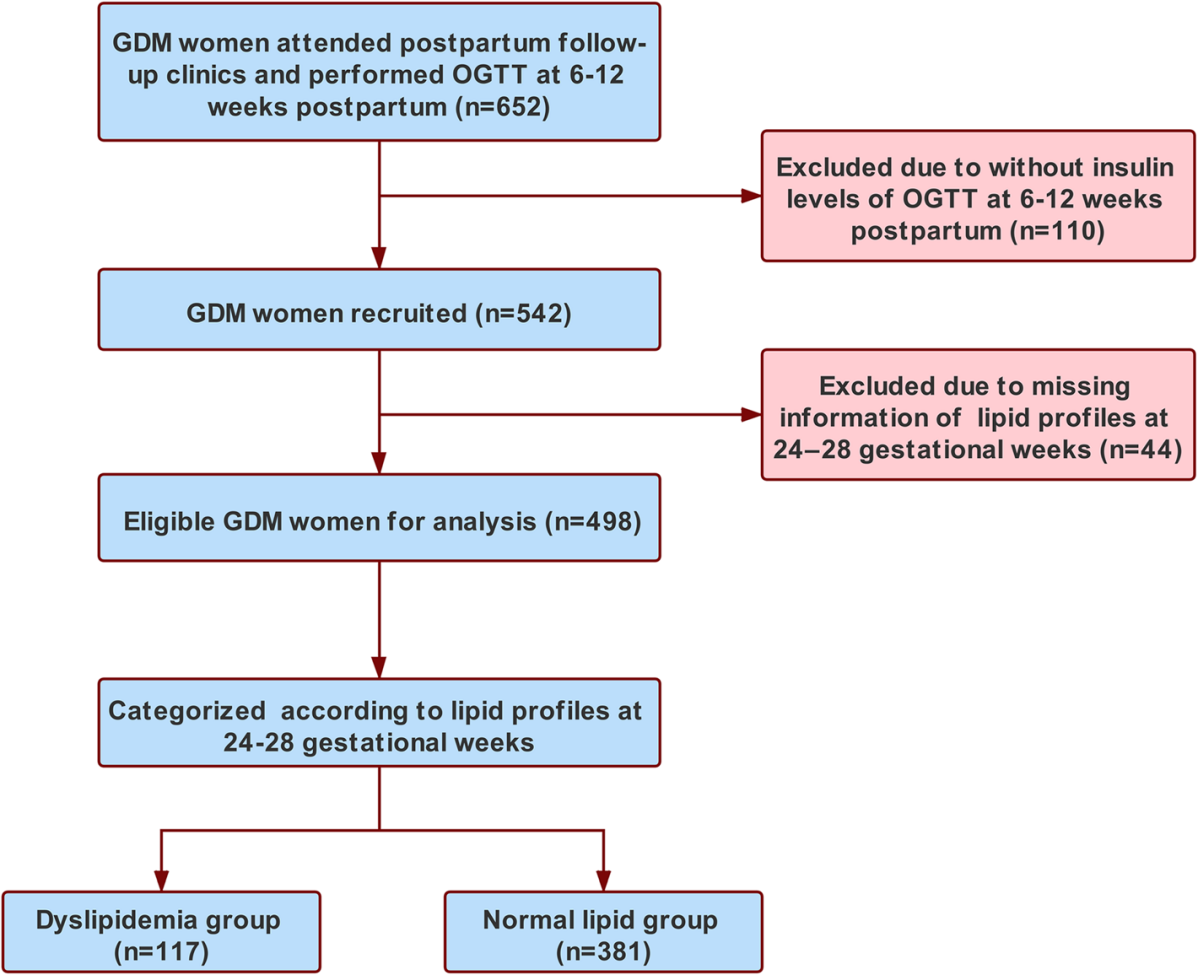
Flowchart of participants included in this study

This was a retrospective cohort study with 652 women who experienced GDM and attended postpartum follow-up clinics at the First Affiliated Hospital, Sun Yat-sen University between January 2015 and December 2018. In total, 110 women for whom plasma insulin concentrations were not assayed in blood samples from the OGTT at 6–12 weeks of postpartum and 44 women for whom plasma lipid concentrations were not measured during 24–28 weeks of gestation were excluded. The remaining 498 women were included in the final analysis (Fig. 1). All the eligible women were categorized into normal lipid group and dyslipidemia group according to their lipid profile at 24–28 weeks of gestation.

### Data collection and metabolic measurements

The following maternal data were collected from medical records: age at delivery, first-degree family history of diabetes, and whether insulin therapy was administered during pregnancy. A 75-g OGTT and plasma lipid measurements were performed at 24–28 weeks of gestation and 6–12 weeks of postpartum. Fasting blood samples for the measurement of plasma lipid concentrations were withdrawn from an antecubital vein after an overnight fast of at least 8 h. Between 24 and 28 gestational weeks, fasting plasma glucose (FPG), 1-h plasma glucose (1hPG), and 2-h plasma glucose (2hPG) were performed during a 75-g OGTT. In the early postpartum 75-g OGTT, the levels of plasma glucose and serum insulin were measured at fasting, 30 min and 2 h after the ingestion of the glucose formulation. All blood samples were analyzed in the laboratory of the Department of Biochemistry of the First Affiliated Hospital, Sun Yat-sen University.

Pancreatic β-cell function was estimated using the 30-min insulinogenic index (IGI_30_), which was calculated using the plasma insulin and glucose concentrations at 0-min and 30-min of the OGTT [11], and homoeostasis model assessment of β-cell function (HOMA-β) [12]. IGI_30_ was calculated as (insulin_60min_ − insulin_0min_ [µU/mL]) / (glucose_30min_ − glucose_0min_ [mmol/L]) and HOMA-β was calculated as 20 × (fasting insulin [µU/mL]) × (fasting glucose − 3.5 [mmol/L]). Insulin sensitivity was assessed using the composite (Matsuda) insulin sensitivity index (ISI) [13] and the homoeostasis model assessment of insulin resistance (HOMA-IR) index [12]. The composite ISI was calculated using the following formula: 10,000 / √{(fasting glucose [mg/dL]) × (fasting insulin [µU/mL]) × (mean glucose [mg/dL]) × (mean insulin [µU/mL])}. HOMA-IR was calculated as (fasting glucose [mmol/L]) × (fasting insulin [µU/mL]) / 22.5. Disposition index (DI) was estimated by multiplying IGI_30_ with composite ISI to reflect β-cell function adjusting for the insulin sensitivity [14].

### Definitions of GDM, dyslipidemia, and AGM

GDM was diagnosed according to “International Association of Diabetes and Pregnancy Study Groups” criteria [15], when any serum glucose value equaled or exceeded the appropriate threshold values during the OGTT performed between 24 and 28 weeks of gestation: FPG: 5.1 mmol/L; 1hPG: 10.0 mmol/L; and 2hPG: 8.5 mmol/L.

Dyslipidemia during the mid-trimester of pregnancy was defined using the recommended reference values for maternal serum lipid profiles in China: total cholesterol (TC) ≥ 7.50 mmol/L, triglyceride (TG) ≥ 3.56 mmol/L, high-density lipoprotein cholesterol (HDL-c) ≤ 1.41 mmol/L, and low-density lipoprotein cholesterol (LDL-c) ≥ 4.83 mmol/L [16].

All the women with GDM were advised to undergo another OGTT at 6–12 weeks of postpartum. AGM was diagnosed according to the guidelines of the Chinese Diabetes Society (2017 edition): FPG ≥ 6.1 mmol/L or 2hPG ≥ 7.8 mmol/L (including impaired fasting glucose [IFG], 6.1 mmol/L ≤ FPG < 7.0 mmol/L; impaired glucose tolerance (IGT), 7.8 mmol/L ≤ 2hPG < 11.0 mmol/L; and T2DM, FPG ≥ 7.0 mmol/L or 2hPG ≥ 11.1 mmol/L) [17].

### Statistical analysis

Nonnormally distributed continuous variables were presented as median with interquartile range (IQR), and categorical variables were expressed as number (percentage). The Mann-Whitney U-test for continuous variables and the chi-square test for categorical variables were used to identify differences between normal lipid group and dyslipidemia group. Logistic regression analysis was used to determine whether abnormal TC, TG, HDL-c, and LDL-c concentrations during the middle trimester of pregnancy were independently associated with early postpartum AGM.

The IGI_30_ and composite ISI threshold values to categorize the participants with postpartum AGM into two groups were determined, respectively. The cut-off values for IGI_30_ and composite ISI were calculated to maximize the Youden Index: max (sensitivity + specificity − 1) [18]. The optimal cut-off for the categorization of IGI_30_ into high and low values was 8.72 and that for ISI was 6.98. All the eligible women who had experienced GDM were categorized into five pathophysiologic subtypes, according to their blood glucose concentration, IGI_30_ categorization, and ISI categorization at 6–12 weeks of postpartum: NGT, AGM-(low IGI_30_ + low ISI), AGM-(low IGI_30_ + high ISI), AGM-(high IGI_30_ + low ISI), and AGM-(high IGI_30_ + high ISI). The Kruskal-Wallis test was used to identify differences among these five groups, and then least-significant difference tests were used for pairwise comparisons between two groups. All the analyses were performed using SPSS 25.0 (IBM Corporation, Armonk, NY, USA). Two-sided *P* values of < 0.05 were considered to represent statistical significance.

## Results

### Baseline characteristics at 24–28 weeks of gestation

On the basis of their lipid profiles at 24–28 weeks of gestation, 117 women with GDM were categorized in the dyslipidemia group and 381 were placed in the normal lipid group. Table 1 showed the comparison of the demographic and metabolic characteristics of women with GDM at 24–28 weeks of gestation in the two groups. The prevalence of insulin therapy during pregnancy in the mid-trimester dyslipidemia group was higher than that in normal lipid group (2.56 % vs. 1.31 %, *P* = 0.009). The women in the dyslipidemia group had higher 1hPG (10.00 [9.20–10.75] mmol/L vs. 9.80 [8.80–10.40] mmol/L, *P* = 0.007), higher 2hPG (9.10 [8.55–9.80] mmol/L vs. 8.80 [8.40–9.40] mmol/L, *P* = 0.008), higher TG (3.03 [2.14–3.83] mmol/L vs. 2.09 [1.73–2.58] mmol/L, *P* < 0.001), and higher LDL-c (4.11 [3.81–4.61] mmol/L vs. 3.31 [2.91–3.69] mmol/L, *P* < 0.001) than the women in the normal lipid group.

**Table 1:** Demographic and metabolic characteristics of GDM women at 24–28 gestational weeks between midtrimester dyslipidemia group and normal lipid group

Variables	Dyslipidemia group (*n* = 117)	Normal lipid group (*n* = 381)	*P* value
Age (years)	34 (31–37)	34 (31–37)	0.543
Family history of diabetes	27 (23.08 %)	85 (22.31 %)	0.862
Prevalence of insulin therapy	3 (2.56 %)	5 (1.31 %)	0.009
FPG (mmol/L)	4.70 (4.30-5.00)	4.60 (4.20-5.00)	0.102
1hPG (mmol/L)	10.00 (9.20-10.75)	9.80 (8.80–10.40)	0.007
2hPG (mmol/L)	9.10 (8.55–9.80)	8.80 (8.40–9.40)	0.008
TC (mmol/L)	6.80 (5.90–7.70)	6.60 (5.70–7.40)	0.101
TG (mmol/L)	3.03 (2.14–3.83)	2.09 (1.73–2.58)	< 0.001
HDL-c (mmol/L)	1.98 (1.46–2.32)	1.94 (1.74–2.17)	0.936
LDL-c (mmol/L)	4.11 (3.81–4.61)	3.31 (2.91–3.69)	< 0.001

### Comparisons of the metabolic characteristics at 6–12 weeks of postpartum

After delivery, the incidences of early postpartum T2DM, IGT, and IFG in the mid-trimester dyslipidemia group were higher than that in the normal lipid group. Women in the mid-trimester dyslipidemia group had higher 30-min plasma glucose, higher 2hPG, and higher 2-h plasma insulin of the postpartum OGTT, higher postpartum TG, and higher postpartum LDL-c than those in the normal lipid group. Compared with women in the mid-trimester normal lipid group, those in the dyslipidemia group had lower IGI_30_, lower ISI, and lower DI in the early postpartum period (Table 2).

**Table 2:** Comparisons of the early postpartum metabolic characteristics in women with GDM between mid-trimester dyslipidemia group and normal lipid group

Variables	Dyslipidemia group (*n* = 117)	Normal lipid group (*n* = 381)	*P* value
Postpartum AGM	66 (56.41 %)	118 (30.97 %)	< 0.001
Postpartum T2DM	7 (5.98 %)	9 (2.36 %)	< 0.001
Postpartum IGT	44 (37.61 %)	75 (19.69 %)	< 0.001
Postpartum IFG	7 (5.98 %)	20 (5.25 %)	0.106
Postpartum IGT + IFG	8 (6.84 %)	14 (3.67 %)	< 0.001
FPG (mmol/L)	4.90 (4.40–5.40)	4.80 (4.30–5.40)	0.154
30 min PG (mmol/L)	8.90 (8.30–9.70)	8.70 (8.20–9.50)	0.008
2hPG (mmol/L)	7.30 (6.00-8.80)	7.10 (5.85–8.60)	0.012
FINS (µU/mL)	5.75 (3.84–9.01)	5.35(3.65–8.27)	0.206
30 min INS(µU/mL)	38.94 (28.43–59.58)	37.55 (26.77–59.41)	0.994
2 h INS(µU/mL)	39.25 (21.23–65.12)	33.42 (21.65–52.58)	0.006
IGI_30_	7.55 (4.91–10.40)	11.35 (7.79–16.29)	< 0.001
ISI	7.26 (5.21–11.56)	7.82 (5.25–13.27)	0.023
DI	54.55 (32.73–84.34)	85.52 (57.42-122.61)	< 0.001
HOMA-β	84.90 (62.30-127.91)	88.85 (61.91-135.66)	0.914
HOMA-IR	1.29 (0.79–2.03)	1.13 (0.75–1.77)	0.216
TC (mmol/L)	6.10 (5.60–7.20)	5.50 (4.80–6.10)	0.116
TG (mmol/L)	2.16 (1.74–2.61)	1.96 (1.65–2.58)	0.007
HDL-c (mmol/L)	1.86 (1.67–2.08)	1.81 (1.59–2.02)	0.943
LDL-c (mmol/L)	3.93 (3.33–4.60)	3.30 (2.83–3.78)	< 0.001

### Relationships between abnormal mid-trimester lipid profile and early postpartum AGM

Logistic regression analyses (Table 3) showed that abnormal mid-trimester TG (crude odds ratio [OR] = 1.961, 95 % confidence interval [CI] = 1.177–2.536, *P* = 0.009) and abnormal LDL-c (crude OR = 1.579, 95 % CI = 1.274–1.936, *P* = 0.039) were associated with the presence of early-postpartum AGM. After adjustment for maternal age, insulin therapy, family history of diabetes, and the three OGTT blood glucose values at 24–28 weeks of gestation, and lipid parameters at 6–12 weeks of postpartum, these associations remained significant (mid-trimester abnormal TG: adjusted OR = 1.786, 95 % CI = 1.142–2.425, *P* = 0.031; and abnormal LDL-c: adjusted OR = 1.621, 95 % CI = 1.323–2.051, *P* = 0.035). In contrast, no significant associations were found between the abnormal mid-trimester TC or HDL-c and the presence of early postpartum AGM.

**Table 3:** Logistic regression analysis of the relationships between the abnormal mid-trimester lipid parameters and early postpartum abnormal glucose metabolism

Characteristics^a^	Crude OR	95 % CI	*P* value	Adjusted OR^b^	95 % CI	*P* value
Abnormal mid-trimester TC for postpartum AGM	0.549	0.219–1.379	0.202	1.079	0.673–1.551	0.152
Abnormal mid-trimester TG for postpartum AGM	1.961	1.177–2.536	0.009	1.786	1.142–2.425	0.031
Abnormal mid-trimester HDL-c for postpartum AGM	0.397	0.174–0.903	0.321	1.080	0.814–1.317	0.126
Abnormal mid-trimester LDL-c for postpartum AGM	1.579	1.274–1.936	0.039	1.621	1.323–2.051	0.035

^a^The reference values for all comparisons were those of the normal mid-trimester lipid parameters

^b^Adjustments were carried out for maternal age, family history of diabetes, insulin therapy and the three OGTT blood glucose values at 24–28 weeks of gestation, and the lipid parameters during early postpartum period

### Comparison of mid-trimester lipid profile among women with different early postpartum glucose metabolism subtypes

**Fig. 2:**
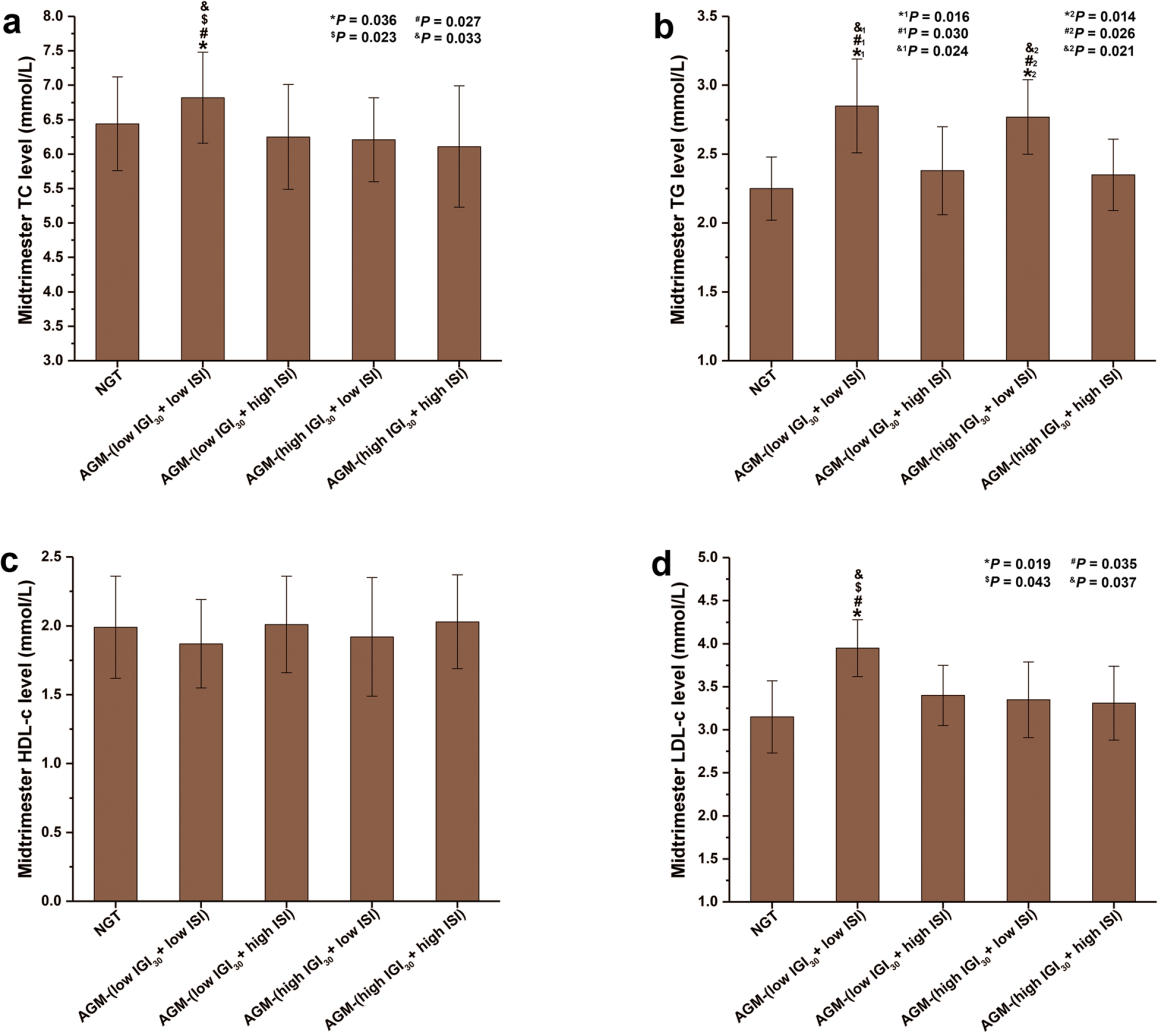
Mid-trimester lipid profile among women with different glucose metabolism subtypes at 6–12 weeks postpartum. **a** Mid-trimester TC concentrations among different glucose metabolism subtypes at 6–12 weeks postpartum. **b** Mid-trimester TG concentrations among different glucose metabolism subtypes at 6–12 weeks postpartum. **c** Mid-trimester HDL-c concentrations among different glucose metabolism subtypes at 6–12 weeks postpartum. **d** Mid-trimester LDL-c concentrations among different glucose metabolism subtypes at 6–12 weeks postpartum. NGT: *n* = 302; AGM-(low IGI_30_ + low ISI): *n* = 43; AGM-(low IGI_30_ + high ISI): *n* = 65; AGM-(high IGI_30_ + low ISI): *n* = 61; AGM-(high IGI_30_ + high ISI): *n* = 27. *P < 0.05 vs. the NGT group; ^#^*P* < 0.05 vs. the AGM-(low IGI_30_ + high ISI) group; ^$^*P* < 0.05 vs. the AGM-(high IGI_30_ + low ISI) group; ^&^*P* < 0.05 vs. the AGM-(high IGI_30_ + high ISI) group

After delivery, the prevalences of NGT, AGM-(low IGI_30_ + low ISI), AGM-(low IGI_30_ + high ISI), AGM-(high IGI_30_ + low ISI), and AGM-(high IGI_30_ + high ISI) were 60.64 % (302/498), 8.63 % (43/498), 13.05 % (65/498), 12.25 % (61/498), and 5.42 % (27/498). Compared with women with NGT or other subtypes of AGM, those with AGM-(low IGI_30_ + low ISI) women had higher mid-trimester TC and LDL-c concentrations (both *P* < 0.05) (Fig. 2a, d). Women with AGM-(low IGI_30_ + low ISI) or AGM-(high IGI_30_ + low ISI) had higher mid-trimester TG concentrations than women with NGT, AGM-(low IGI_30_ + high ISI), or AGM-(high IGI_30_ + high ISI) (*P* < 0.05) (Fig. 2b). No significant differences were found in mid-trimester HDL-c concentrations among the women with postpartum NGT or one of the four subtypes of AGM (Fig. 2c).

## Discussion

In the present study, the incidence of early postpartum AGM in GDM women with mid-trimester dyslipidemia was significantly higher than that with normal lipid during midterm pregnancy. In particular, GDM women with abnormal mid-trimester TG and LDL-c concentrations were predisposed to early postpartum AGM. An elevated mid-trimester TG concentration was associated with low insulin sensitivity in postpartum women with AGM who had previously experienced GDM. Furthermore, GDM women who progressed to early postpartum AGM, low insulin secretion, and low insulin sensitivity had higher mid-trimester TC and LDL-c concentrations.

The incidence of postpartum AGM in women with a history of GDM had been reported to vary between 2.6 and 38 % within 6–12 weeks of delivery [19, 20]. In the present study, the incidence of early postpartum AGM in women who had experienced GDM and had mid-trimester dyslipidemia was 56.41 %, which was significantly higher than the incidence in those with normal lipid concentrations (30.97 %). The serum concentrations of lipid parameters (TC, TG, LDL-c, and HDL-c) gradually increase from 12 weeks of pregnancy and show more marked increases during the second and third trimesters [21]. Numerous studies showed that serum lipid abnormalities were associated with disturbances in glucose metabolism [22, 23].

This study showed that GDM women with abnormal mid-trimester TG was predisposed to early postpartum AGM, which was consistent with the results of another retrospective study that was conducted in China [24]. A high TG was a lipid abnormality that commonly accompanies T2DM and prediabetic states [25]. Previous studies showed that the elevated TG concentration was an independent predictor of the development of diabetes in middle-aged women [26] and TG was a risk factor for postpartum glucose intolerance [27]. One potential link between TG and disturbances in glucose metabolism was that TG may induce insulin resistance. In the present study, we found that GDM women with postpartum AGM and low insulin sensitivity had high mid-trimester TG concentrations. Free fatty acids (FFAs) derived from the high TG might mediate a vicious cycle between TG and insulin resistance [23, 28]. Excess FFAs concentrations might result in the generation of toxic lipid species, including diacylglycerides and ceramides. These toxic lipids contributed to endoplasmic reticulum stress, mitochondrial dysfunction, and the generation of reactive oxygen species, which together induced inflammation and insulin resistance [29, 30]. Furthermore, FFAs affected the fatty acid composition of cellular membranes, which directly affected cellular function, as well as the incorporation of insulin receptors into membranes [31]. Thus, the higher concentration of TG during pregnancy may have induced subsequent postpartum AGM mainly via FFAs.

Elevated plasma TC concentration was always recognized as an independent risk factor for metabolic syndrome, diabetes, and coronary heart disease [32]. Furthermore, a large retrospective study that showed that the TC concentration during early pregnancy was an independent risk factor for GDM [33]. However, in the present study, mid-trimester TC was not significantly associated with postpartum AGM in women who had previously experienced GDM, whereas those with postpartum AGM, low insulin secretion, and low insulin sensitivity had high mid-trimester TC concentrations. This finding implied that the plasma TC concentration differed among women with distinct pathophysiologic subtypes of postpartum AGM, which may not be apparent when investigating AGM as a single group.

A previous study revealed that a high concentration of LDL-c in women with GDM was an independent risk factor for insulin resistance after delivery [34]. However, another study showed no significant difference in the LDL-c concentrations of postpartum women with NGT or AGM and a history of GDM [35]. The findings of this study were consistent with a positive association between LDL-c concentration and the prevalence of postpartum AGM. The underlying mechanisms of the associations between LDL-c and postpartum AGM were not fully understood. Some studies demonstrated that abnormal LDL-c metabolism might lead to the loss of function of pancreatic β-cells [36] and excessive LDL-c accumulation might aggravate oxidative stress, which correlated with insulin resistance [34, 37]. Consistent with this, the present study showed that GDM women with postpartum AGM, low insulin secretion, and low insulin sensitivity had elevated mid-trimester LDL-c concentrations. High LDL-c concentrations during the middle of pregnancy among GDM women were associated with both β-cell dysfunction and insulin resistance after delivery.

Oxidative stress [38] and chronic inflammation [39] are involved in the progression of GDM. There was a large body of research concerning the role of plasma HDL-c as a protective agent. HDL-c had anti-oxidant, anti-thrombotic, anti-inflammatory, vasodilatory effects, and a protective function against endothelial cell damage [40–42]. High concentrations of TG, TC, and LDL-c were common in pregnant women and especially associated with GDM [7, 33], whereas HDL-c did not change significantly during gestation even in GDM women [43]. This was similar to this study. In the present study, no significant differences were found in mid-trimester HDL-c concentrations between GDM women with postpartum AGM and those with NGT. Further investigation is needed to determine whether mid-trimester HDL-c is a protective factor for postpartum AGM.

### Study strength and limitations

In the present study, the relationship between lipid profile during the mid-trimester and the pathophysiologic subtype of early postpartum abnormal glucose metabolism in women who experienced GDM was analyzed for the first time. This study also had several limitations. First, although the maternal lipid profile in the middle trimester (24–28 weeks of gestation) was assessed, the precise data of gestational weeks were not recorded. Lipid concentrations may change constantly through gestational weeks. Second, the body weight information of most GDM women before or during pregnancy was missing, limiting the possibility of analyzing the effect of overweight or obese on early postpartum AGM. Third, lipid profile may be closely associated with lifestyle. However, the information regarding the lifestyles of the participants was not collected, such as dietary factors and physical activity, which could be confounders. Finally, this was a hospital-based retrospective study and the sample lacked diversity. Therefore, further large multi-center studies with more rigorous design are needed in the future.

## Conclusions

In conclusion, GDM women with abnormal mid-trimester TG and LDL-c concentrations were predisposed to early postpartum AGM. Postpartum AGM women who experienced GDM had heterogeneous mid-trimester lipid profile according to their pathophysiologic subtype. Among these postpartum AGM women, those with low insulin sensitivity had elevated mid-trimester TG concentrations, while those with low insulin secretion and low insulin sensitivity had higher mid-trimester TC and LDL-c concentrations. Thus, more attention should be paid to the mid-trimester lipid profile in women with GDM. For these women with abnormal lipid profile during midterm pregnancy, a low-fat diet, lifestyle modifications, and intensive follow-up are strongly recommended for the prevention of postpartum AGM.

## Data Availability

All data used in this study are available from the corresponding author upon reasonable request.

## Abbreviations

AGM: Abnormal glucose metabolism
CI: Confidence interval
DI: Disposition index
FFAs: Free fatty acids
FPG: Fasting plasma glucose
GDM: Gestational diabetes mellitus
HDL-c: High-density lipoprotein cholesterol
HOMA-β: Homoeostasis model assessment of β-cell function
HOMA-β: Homoeostasis model assessment of insulin resistance
IFG: Impaired fasting glucose
IGI_30_: 30-min insulinogenic index
IGT: Impaired glucose tolerance
IQR: Interquartile range
ISI: Insulin sensitivity index
LDL-c: Low-density lipoprotein cholesterol
NGT: Normal glucose tolerance
OGTT: Oral glucose tolerance test
OR: Odds ratio
TG: Triglyceride
TC: Total cholesterol
1hPG: 1-h plasma glucose
2hPG: 2-h plasma glucose
